# Pandemic Strain of Foot-and-Mouth Disease Virus Serotype O

**DOI:** 10.3201/eid1112.050908

**Published:** 2005-12

**Authors:** Nick J. Knowles, Alan R. Samuel, Paul R. Davies, Rebecca J. Midgley, Jean-François Valarcher

**Affiliations:** * Institute for Animal Health, Pirbright, United Kingdom

**Keywords:** Aphthovirus, Epidemiology, Molecular, Foot-and-Mouth Disease Virus, Molecular Sequence Data, Phylogeny, Picornaviridae, Research

## Abstract

A particular genetic lineage of foot-and-mouth disease virus (FMDV) serotype O, which we have named the PanAsia strain, was responsible for an explosive pandemic in Asia and extended to parts of Africa and Europe from 1998 to 2001. In 2000 and 2001, this virus strain caused outbreaks in the Republic of Korea, Japan, Russia, Mongolia, South Africa, the United Kingdom, Republic of Ireland, France, and the Netherlands, countries which last experienced FMD outbreaks decades before (ranging from 1934 for Korea to 1984 for the Netherlands). Although the virus has been controlled in all of these normally FMD-free or sporadically infected countries, it appears to be established throughout much of southern Asia, with geographically separated lineages evolving independently. A pandemic such as this is a rare phenomenon but demonstrates the ability of newly emerging FMDV strains to spread rapidly throughout a wide region and invade countries previously free from the disease.

## Abstract 2

The PanAsia strain is spreading explosively in Asia and extending to parts of Africa and Europe.

Foot-and-mouth disease virus (FMDV, family *Picornaviridae*, genus *Aphthovirus*) causes an acute vesicular disease of pigs and wild and domesticated ruminants such as cattle, water buffalo, sheep, goats, and deer (*1*). It can cause high death rates in young animals and production losses in adults and is considered to be the single most important constraint to world trade in live animals and animal products. Spread of FMDV is predominantly associated with the legal and illegal movement of infected animals or their products.

The Food and Agriculture Organization World Reference Laboratory for Foot-and-Mouth Disease (WRLFMD) is established within the high-security laboratory at the Institute for Animal Health, Pirbright, United Kingdom (*2*). From 2000 to 2004, WRLFMD received an annual average of 536 samples to diagnose FMD from regions of the world where the disease is endemic, predominantly Africa and Asia. Seven serotypes of FMDV exist: SAT 1, SAT 2, and SAT 3 are usually restricted to Africa; Asia 1 is restricted to Asia; and O, A, and C are present in Africa, Asia, and South America and occasionally Europe. In each of the last 5 years, serotype O has been isolated from >60% of the positive FMD samples received.

The economic consequences of FMD incursion into disease-free regions may be severe. For instance, in the first 3 months of the 1997 outbreak in Taiwan, >6,000 farms were affected, 4 million pigs were destroyed or died from the disease, and >21 million doses of vaccine were used (*3*). The cost of controlling the disease was estimated at US $378.6 million. An additional $1.6 billion was lost in export trade, and >65,000 jobs in pig farming and associated industries were lost (*3*). To control the FMD outbreak without using vaccination, animals were slaughtered on >10,000 farms in the United Kingdom in 2001; only one fifth of these animals were actually infected. Four million animals were slaughtered for control measures and 2.5 million more for animal health reasons (*4*). The direct and indirect losses were estimated at ≈£8 billion (*5*).

FMDV has a genome consisting of a single strand of positive-sense RNA. Consequently, the virus has a high mutation rate and may change, on a random basis, 1–8 nucleotides (nt) per replication cycle (*6*). Nucleotide sequencing of part or all of the genome region coding for the outer capsid polypeptide VP1 was first used to study the epidemiology of FMD by Beck and Strohmaier (*7*), who investigated the origin of outbreaks of types O and A in Europe over a 20-year period. Since then, genetic variability has been used to individually characterize strains of FMDV and track their movement across international borders (*8*), and a large number of epidemiologic studies have been published (*9*). Previously, on the basis of comparisons of partial VP1 sequences (≈170 nt at the 3´ end of the gene) of FMD type O viruses, differences between 2 isolates within 4% have been suggested to indicate a recent common origin, whereas differences of >15% signify geographic isolation over many years (*10*), similar to the distinctions made between human polioviruses (*11*). Isolates with >85% nt sequence identity have been placed within groups or topotypes, which tend to be restricted in their geographic distribution (*10**,**12*). The 10 topotypes have been named Europe-South America (Euro-SA), Middle East–South Asia (ME-SA), Southeast Asia (SEA), Cathay (CHY), West Africa (WA), East Africa 1 (EA-1), East Africa 2 (EA-2), East Africa 3 (EA-3), Indonesia-1 (ISA-1), and Indonesia-2 (ISA-2). The Indonesian topotypes, which have not been identified since 1983, are considered extinct.

Knowles et al. (*13*) described the emergence and spread of the PanAsia strain from 1990 to 2000 on the basis of comparisons of partial (and some complete) VP1 sequences from 60 virus isolates. This article extends the molecular epidemiology of this virus strain by comparing 188 complete VP1 sequences for FMD type O viruses mostly isolated from 2000 to 2005 with published sequences of selected viruses from the previous decade and some reference virus strains (N = 151).

## Materials and Methods

### Viruses and Primers

The designation and origin of FMDV isolates studied are listed in Table A1.

Three alternative primer combinations were used for reverse transcription–polymerase chain reaction (RT-PCR): O-1C244F/NK61, O-1C272F/NK61, and O-1C283F/NK61, which have amplicon sizes of 1,181, 1,153, and 1,142 bp, respectively (Table). Forward and reverse primer amounts were 20 and 40 pmol, respectively. We used 4–6 internal sequencing primers to ensure coverage of the VP1 region on both DNA strands (Table).

**Table:** Oligonucleotide primers used for RT-PCR and cycle sequencing of FMDV strains*

Primer	Primer sequence (5´ → 3´)	Sense	Location on FMDV genome		Use
			Gene	Position†	
ARS4	ACCAACCTCCTTGATGTGGCT	+	1C	2349–2369	RT-PCR
O-1C244F	GCAGCAAAACACATGTCAAACACCTT	+	1C	2469–2494	RT-PCR
O-1C272F	TBGCRGGNCTYGCCCAGTACTAC	+	1C	2497–2519	RT-PCR
O-1C283F	GCCCAGTACTACACACAGTACAG	+	1C	2508–2530	RT-PCR
NK61	GACATGTCCTCCTGCATCTG	–	2B	3630–3649	RT-PCR
NK72	GAAGGGCCCAGGGTTGGACTC	–	2A/2B	3558–3578	Sequencing
O-1C499F	TACGCGTACACCGCGTC	+	1C	2724–2740	Sequencing
O-1C583F	GACGGYGAYGCICTGGTCGT	+	1C	2808–2827	Sequencing
A-1C612F	TAGCGCCGGCAAAGACTTTGA	+	1C	2834–2854	Sequencing
O-1D296F	ACAACACCACCAACCCAAC	+	1D	3181–3199	Sequencing
O-1D628R	GTTGGGTTGGTGGTGTTGT	–	1D	3181–3199	Sequencing

*RT-PCR, reverse transcription–polymerase chain reaction; FMDV, food-and-mouth disease virus.

†Position on the genome of O1/Kaufbeuren/FRG/66 (EMBL/GenBank accession no. X00871).

### RT-PCR of vRNA

Total RNA was extracted from 460 μL of a 10% epithelial suspension or cell culture supernatant by using RNeasy kits (Qiagen Ltd., Crawley, West Sussex, UK), according to the manufacturer's instructions, and resuspended in 50 μL nuclease-free water. This RNA (5 μL) was used as the template in a 1-step RT-PCR (Ready-To-Go RT-PCR Beads; Amersham Pharmacia Biosciences, Chalfont St. Giles, Bucks, UK). The following thermal profile was used: 42°C for 30 min; 94°C for 5 min; 35 cycles of 94°C for 60 s; 60°C for 60 s; and 72°C for 90 s; followed by a final extension of 72°C for 5 min. PCR products were analyzed by electrophoresis on a 1.5% agarose-Tris-borate-EDTA gel containing 0.5 μg/mL ethidium bromide. DNA weight markers (GeneRuler 100 bp DNA Ladder Plus, Ready-To-Use; Fermentas, Inc., Hanover, MD, USA) were run alongside the samples to facilitate product identification and quantification. Post-PCR removal of deoxynucleoside triphosphates and primers was achieved enzymatically by using ExoSAP-IT (USB Corporation, Cleveland, OH, USA), according to the manufacturer's instructions.

### Sequence Determination

PCR amplicons were sequenced by using the DTS Quick Start Kit (Beckman Coulter Inc., Fullerton, CA, USA) according to the manufacturer's instructions and with the sequencing primers listed in the Table. The sequencing reactions were run on a CEQ8000Automated Sequencer (Beckman Coulter) according to the manufacturer's instructions. The sequences determined in this study have been submitted to the EMBL/GenBank/DDBJ databases; accession numbers are shown in Table A1.

### Phylogenetic Analysis

An unrooted neighbor-joining tree was constructed by using MEGA version 3 (*14*). The robustness of the tree topology was assessed with 1,000 bootstrap replicates as implemented in the program.

## Results and Discussion

Virus RNA was extracted from 188 FMD type O viruses, and each VP1-coding region was successfully amplified by RT-PCR by using at least 1 of the 3 described primer sets. The complete VP1 sequences were determined by directly sequencing the amplicons. For all these isolates, the VP1 gene consisted of 633 nt coding for 211 amino acids (previously VP1 was considered to be 2 amino acids longer at its carboxyl-terminus; however, the VP1-2A cleavage site is actually between a conserved glutamine [VP1^211^ in most type Os] and a variable residue [2A^1^, often a leucine in serotype O]) (*15*).

**Figure 1:**
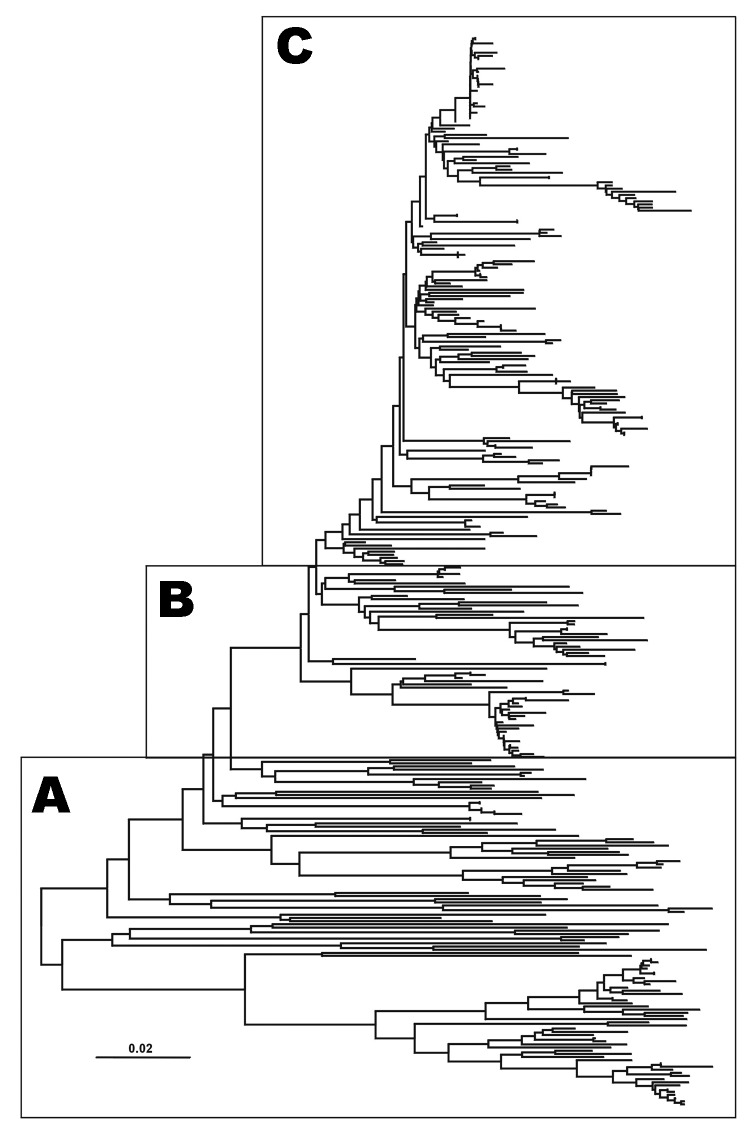
Midpoint–rooted neighbor-joining tree showing the relationships between the 339 VP1 sequences studied. Only the tree structure is shown; details of the boxes labeled A to C are shown in Figures 2–4.

**Figure 2:**
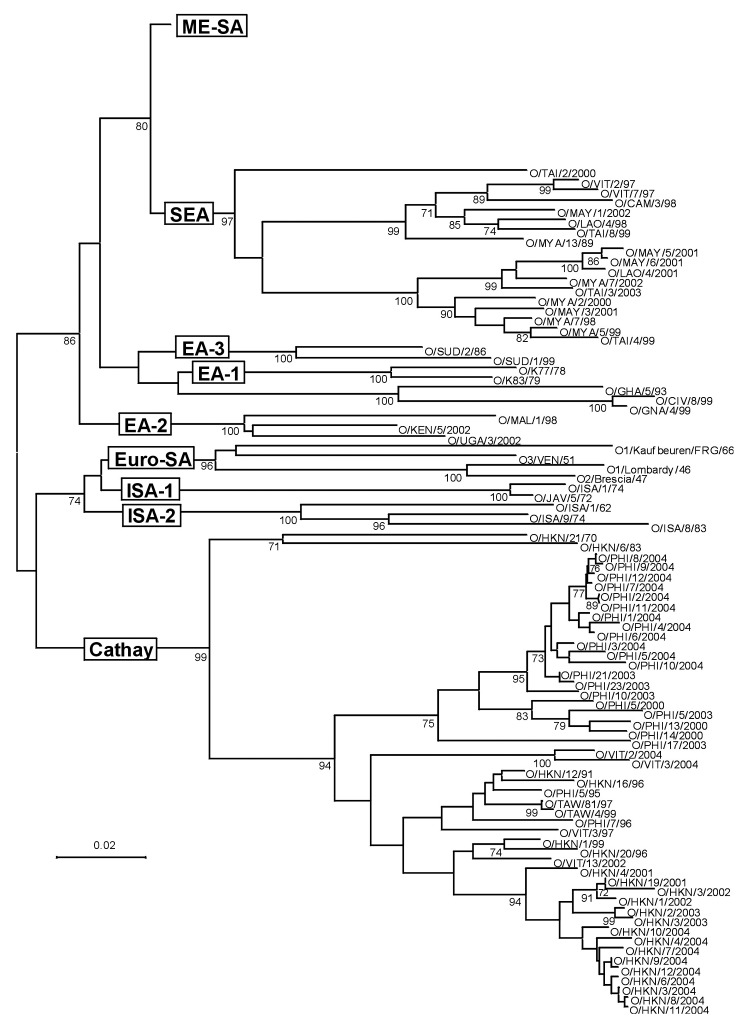
Midpoint-rooted neighbor-joining tree showing the Cathay, Europe-South America (Euro-SA), Indonesia-1 (ISA-1), Indonesia-2 (ISA-2), West Africa (WA), East Africa 1 (EA-1), East Africa 2 (EA-2), and East Africa 3 (EA-3) topotypes. Only bootstrap values >70% are shown.

**Figure 3:**
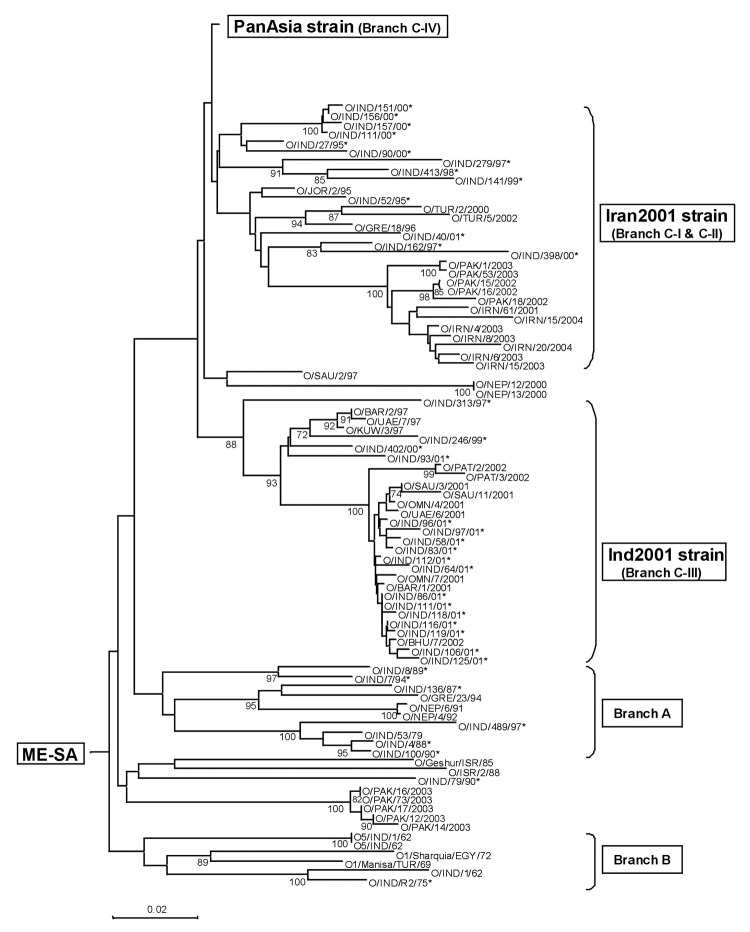
Midpoint-rooted neighbor-joining tree showing the Middle East–South Asia (ME-SA) topotype (except the PanAsia strain). Only bootstrap values >70% are shown.

**Figure 4:**
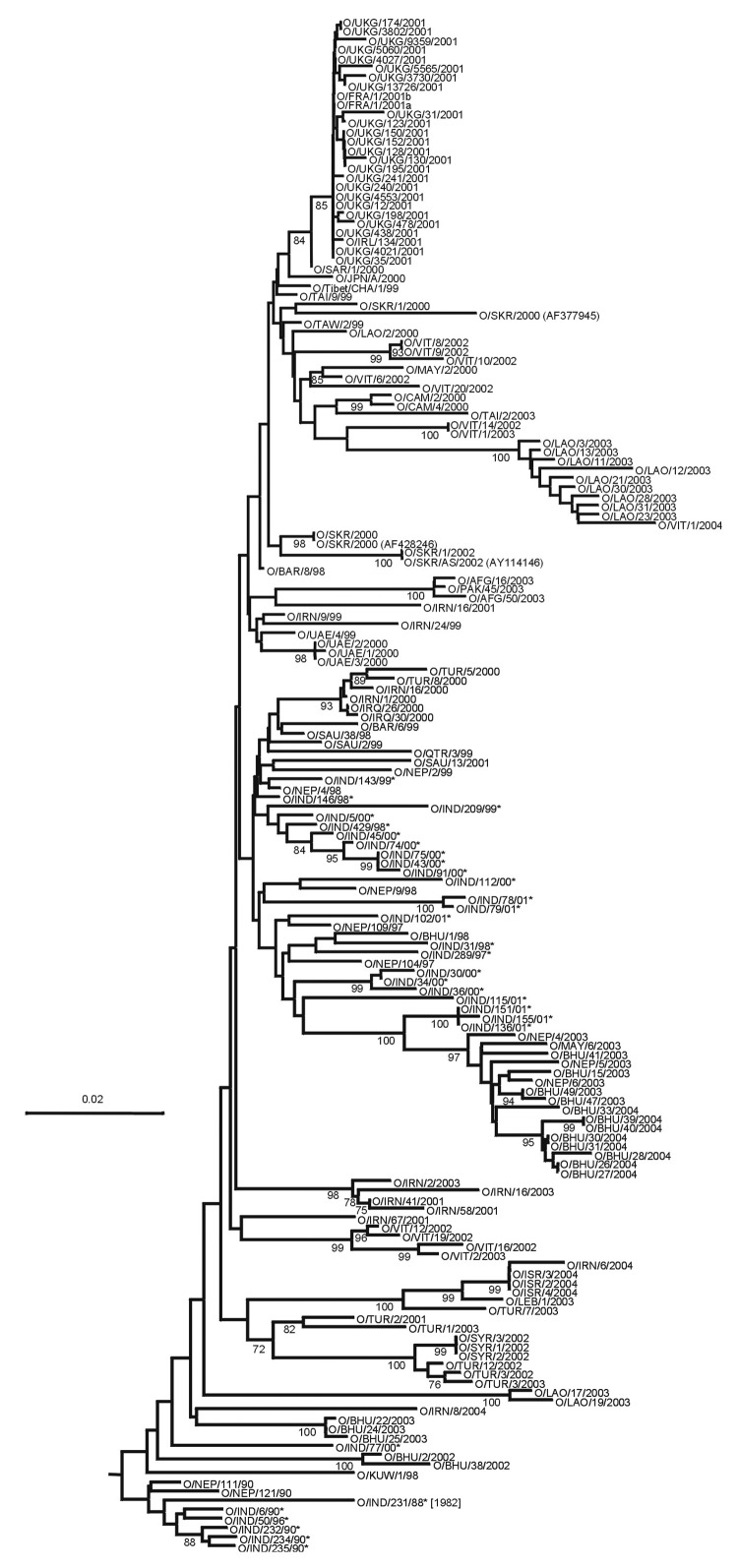
Midpoint-rooted neighbor-joining tree showing the PanAsia strain. Only bootstrap values >70% are shown.

The 188 VP1 sequences we report were compared to 151 VP1 sequences previously published or awaiting publication (database accession numbers are listed in Table A1). A bootstrapped neighbor-joining tree containing all 339 sequences was constructed by using MEGA 3 (Figure 1). Figures 2–4 show various parts of the tree depicted in Figure 1 in greater detail. The bootstrap support for the 10 FMDV O topotypes was generally high (96%–100%; Figure 2). The topotype distributions of the 299Asian FMD type O viruses (including those reported elsewhere) were as follows: ME-SA (253), SEA (18), and Cathay (49) (Table A1). Additionally, 26 European viruses (from the United Kingdom, Ireland, and France) belonged to the ME-SA topotype. The PanAsia strain accounted for 168 (66%) of the 253 ME-SA isolates.

Some FMDV O topotypes had a more limited spread than the ME-SA topotype. Virus isolates from Hong Kong and the Philippines all fell within the Cathay topotype; all the recently isolated (2000–2004) Philippines isolates form a distinct lineage. This topotype was first introduced into the Philippines in 1994, probably from mainland China or Hong Kong (the only known places where it existed at that time). Earlier isolates from the Philippines (e.g., O/PHI/5/95) were closely related to Hong Kong viruses (Figure 2). This topotype was first seen in Vietnam in 1997 and continued to occur there until 2004 (Figure 2) but has not, as far as we know, spread to neighboring Southeast Asian countries. A Cathay topotype virus also spread to Taiwan in 1997, where it caused an extensive epidemic that lasted until at least 1999 (*3*) (Figure 2). Viruses belonging to the SEA topotype continue to be isolated throughout Southeast Asia (Figure 2; Table A2), despite the recent introduction and widespread dissemination of the PanAsia strain. No examples of either of the Indonesian topotypes have been detected in the field since 1983.

Viruses belonging to the ME-SA topotype occur in many genetic sublineages (Figure 3). These were often initially found in India and subsequently spread to other geographic regions. The reference/vaccine strains (O_5_/IND/1/62, O_1_/Manisa/TUR/69, O_1_/Sharquia/EGY/72, and O/IND/R2/75) all occur in a single lineage distinct from later isolates. The O_5_/IND/1/62 sequenced by Hemadri et al. (*16*) is different (9.6%) from the same strain that we and others sequenced (*17**,**18*) (all 3 sequences are identical, and the virus stocks probably all originated from WRLFMD), and the origin of these isolates requires further investigation. Two other reference/vaccine strains (O/Geshur/ISR/85 and O/Dalton/ISR/2/88) fall on another lineage but are not closely related to each other. Within the ME-SA topotype, several sublineages have been defined as strains, such as PanAsia, Ind2001, and Iran2001, on the basis of phylogenetic relationships and a nucleotide difference of <5% (*9**,**16*). However, these are artificial groupings, the edges of which become blurred as viruses evolve in different directions. For example, the nucleotide sequences of 2 viruses that are on the PanAsia lineage, O/VIT/1/2004 and O/BHU/27/2004, differ from O/TAW/2/99 by 5.4% and 5.0%, respectively, but differ from each other by 7.9%. Thus trying to define "strains," particularly using percentage nucleotide relationships, may not be relevant, except in special circumstances, such as a pandemic caused by a cluster of closely related viruses.

Viruses that we consider part of the PanAsia strain (within the ME-SA topotype) are shown in Figure 4. Within the PanAsia strain, different sublineages can be distinguished despite some low bootstrap values. Some of them correspond to well-defined geographic areas in which these isolates have been collected through the years and show evolutionary relationships. Others are mixtures of FMDV isolates from different regions. In such cases, the phylogeny gives clues to the probable source of some isolates. The PanAsia strain shows a limited degree of variability of the VP1 gene during the outbreak in 2001 in the United Kingdom. Indeed, the degree of genetic variability of the VP1 gene of 24 isolates collected between the beginning and the end of the outbreak was <1.29%, and very few amino acid changes were observed (a maximum of 3 in any 1 sequence).

According to our current analysis, the PanAsia strain is an emergent sublineage of FMDV that, after several years in India, spread through southern Asia, the Middle East, and Europe. This strain apparently was confined to India for longer—and then spread much faster—than previously believed. In 1994, Samuel et al. (*19*) first noted the arrival of a new FMDV type O lineage in Saudi Arabia. Previously, we had considered this lineage to be part of the PanAsia strain (*13*). However, analysis of complete VP1 sequences with the neighbor-joining algorithm, rather than unweighted pair-group method analysis on partial VP1 sequences, indicated that these viruses, along with others isolated between 1994 and 1997 in Asia (except India), actually belong to 1 of 2 distinct lineages that we have termed Ind2001 and Iran2001 (Figure 3). Therefore, viruses that we would now classify as PanAsia first appeared in Bahrain, Iran, Lebanon, Kuwait, Saudi Arabia, and Yemen much later (i.e., in 1998); in Israel, Turkey, and the United Arab Emirates in 1999; and in Malaysia in 2000 (Figures 3 and 4; data not shown). In Nepal in 1990, viruses were found that were closely related to the earliest PanAsia isolates from India in the same year. However, from 1991 to 1996, only viruses belonging to non-PanAsia lineages of ME-SA were found in Nepal. During the years 1997–1999, PanAsia viruses were once again found. This virus lineage may have persisted in Nepal in the intervening years (since only a few virus isolates have been examined) or may have been reintroduced in 1997. This extension and reanalysis of the sequence data indicate that the spread of the PanAsia strain from the Indian subcontinent was probably more explosive than once thought and principally occurred from 1998 to 2001.

Retrospective examination of viruses from India indicated that the PanAsia strain was present in the north of that country as early as 1990 and may even have been present as far back as 1982 (*16*). From 1991 to 1997, the new lineage appeared to spread to other parts of India (*16*).

The presumed initial spread from India in 1998 was to Bhutan, Bahrain, Iran, Jordan, Kuwait, Lebanon, Syria, Saudi Arabia, and the Yemen Arab Republic. In May 1999, the People's Republic of China reported FMD outbreaks in Tibet, Hainan, and Fujian Provinces (*20*). Sequencing viruses from the outbreaks in Tibet (O/CHA/1/99, O/CHA/2/99, and O/CHA/3/99) and Hainan (O/CHA/4/99) showed that they belonged to the new lineage (*13*) (Figure 4). In June 1999, FMDV was isolated from subclinically infected or carrier cattle in Kinmen Prefecture of Taiwan Province of China (POC) during routine surveillance. Sequence analysis of this isolate (O/TAW/2/99) showed it also belonged to the new lineage (Figure 4). Later that month, FMDV was detected in Tainan Prefecture on the main island of Taiwan, again in cattle showing no signs of disease. In January 2000, the first clinical cases in cattle were found in Taiwan (Yunlin and Chiayii Prefectures) and in February 2000, ≈71 young goats in Kaoshiung and Changhwa Prefectures died suddenly from FMD, although no disease was seen in adult goats that had been vaccinated. The distribution of this sublineage throughout Asia justified its name of the PanAsia strain.

Towards the end of 1999, the PanAsia virus was clearly moving into Southeast Asia (Myanmar, Thailand, Vietnam, Lao People's Democratic Republic) (Table A2), where the FMDV type O SEA topotype had existed exclusively (at least until the Cathay topotype was introduced into Vietnam in 1997) (*10*). By April 2000, all mainland Southeast Asian countries had experienced outbreaks due to the new strain.

In March 2000, FMD type O appeared in South Korea and Japan, and sequence analysis indicated that the PanAsia strain was responsible (*13*) (Figure 4). In April 2000, a severe outbreak of FMD type O in occurred in pigs in the Ussuriysk District of eastern Russia. Of 625 pigs affected, nearly 37% died from the disease. Sequencing the VP1 gene showed that the PanAsia strain was responsible (*13*). At the end of April 2000, an outbreak of FMD type O was reported in Ulaanbadrakh Soum County, Dornogovi Province, Mongolia. In this outbreak sheep, goats, and cattle were affected. Again, sequence analysis of the VP1 gene showed the virus to be of the PanAsia lineage (*13*). In September 2000, the PanAsia strain spread to KwaZulu-Natal Province in South Africa (*13**,**17*) (Figure 4); the origin was traced to feeding pigs with uncooked swill from a ship in the port of Durban (*21*). This FMD outbreak is the first since 1957 in this region of South Africa and the first recorded outbreak in that country due to serotype O. In February 2001, FMD was diagnosed in the United Kingdom; by the end of July, >1,900 farms were affected. The PanAsia strain was responsible for these outbreaks (*13**,**22**,**23*). In late February 2001, the disease spread from the British mainland to Northern Ireland, and in March and April outbreaks of FMD type O were also reported in the Republic of Ireland (n = 1), France (n = 2), and the Netherlands (n = 26). In 2003, the PanAsia strain was detected for the first time in Afghanistan, Nepal, and Pakistan; however, because of lack of samples or sequencing data, the strain may have been present earlier. Since 2003, the PanAsia strain has not been detected in any new countries.

The PanAsia strain has not yet been detected in Africa (except South Africa in 2000) or South America, despite extensive unpublished sequence studies by ourselves; the Onderstepoort Veterinary Institute, South Africa (W. Vosloo, pers. comm.); and the Pan-American FMD Center, Brazil (I.E. Bergmann, pers. comm.). However, the PanAsia strain is present in many countries in which FMD is endemic and occurs in countries in which the incidence of FMD is sporadic.

The extent of this spread is unique for a single strain of FMDV, and its presence in most recent samples from the Middle East indicates that it has dominated and outcompeted the other strains of FMDV previously observed (*19*). While we acknowledge that the sampling of virus isolates is not random (i.e., the samples examined are those submitted to WRLFMD by some of the countries experiencing outbreaks), the same sampling technique has shown a marked increase in the number of isolations of the PanAsia lineage over the preceding years.

The appearance of the PanAsia virus in countries that have been FMD-free for many years shows that this strain is capable of spreading to countries where strict control measures are normally effective at preventing importation of animal pathogens. Whether this fitness to survive is related to particular features of the transmissibility of the virus strain or its ability to spread subclinically in certain breeds of animal, as found in Taiwan in 1999 or in Japan in 2000 (*24*), is not clear. The PanAsia virus strain has been isolated from a wide variety of host species, including cattle, water buffalo, pigs, sheep, goats, and gazelle (Qatar in 1999), and its ability to infect a wide range of species could be a contributing factor in its success. Within the PanAsia strain, differences in behavior of the virus, such as host species or virulence, remain unexplained on a genetic basis, according to comparison of the full genome sequences from viruses from this group (*25*). However, these characteristics can also be biased by practices such as vaccination, the animal population targeted for vaccination, or the animal species that are farmed in a particular area.

We have no evidence of increased or altered trade in the region that could explain the sudden spread of the PanAsia virus. Additionally, the lack of efficacy of existing FMDV vaccines does not seem to be responsible for the spread of this strain in countries in which vaccination is practiced. Indeed, antigenic matching analysis has shown good cross-reactivity between field isolates of the PanAsia strain and current vaccine strains such as O_1_ Manisa (WRLFMD, data not shown), and this finding has been confirmed for O/UKG/2001 virus by cross-protection studies (*26**,**27*).

The spread of the PanAsia strain across most of Asia and into Europe and South Africa demonstrates how a newly evolved virus may become established, in spite of control measures at international borders. FMD in a previously disease-free country can seriously interfere with the local and export trade in susceptible animals and their products. A large outbreak of FMD in northern Europe or the United States could result in losses of several billion US dollars. The emergence of this strain of FMDV, and its spread within the territory bounded by Ireland in the west and Japan in the east, provides an example of the economic damage that can result. It also demonstrates the difficulty of containing such a transmissible virus within a defined region. The emergence of such strains highlights the necessity to constantly monitor and characterize field isolates responsible for outbreaks in FMD-endemic countries and the need for countries to be rapidly alerted so that appropriate control measures can be instituted. For this purpose, an international early warning system must be established to share information on the characteristics of the latest FMDV isolates in real time.

## Appendix

**Table A1:** Designation and origin of food-and-mouth disease type O viruses studied. Download PDF (64 KB, 13 pages)

Topotype	Strain	Virus designation	Geographic origin	Date	Species	Database
ME-SA	PanAsia	O/AFG/16/2003	Tashqurghan, Balkh, Afghanistan	Apr 10, 2003	Bovine	DQ165035
ME-SA	PanAsia	O/AFG/50/2003	Center, Takhar, Afghanistan	Apr 14, 2003	Bovine	DQ165036
ME-SA	Ind2001	O/BAR/2/97	Bahrain	1997	Not known	AJ318824†
ME-SA	PanAsia	O/BAR/8/98	Bahrain	1998	Bovine	AJ318825†
ME-SA	PanAsia	O/BAR/6/99	Bahrain	1999	Bovine	DQ164862†
ME-SA	Ind2001	O/BAR/1/2001	Bahrain	May 2001	Bovine	DQ164863†
ME-SA	PanAsia	O/BHU/1/98	Serbithang, Bhutan	Feb 8, 1998	Porcine	AJ318826†
ME-SA	PanAsia	O/BHU/2/2002	Bhutan	2002	Not known	DQ165037
ME-SA	Ind2001	O/BHU/7/2002	Bhutan	2002	Not known	DQ164864†
ME-SA	PanAsia	O/BHU/38/2002	Bumthang, Bhutan	Jul 29, 2002	Bovine	DQ165038
ME-SA	PanAsia	O/BHU/15/2003	Eastern Region, Bhutan	Aug 6, 2003	Bovine	DQ164865†
ME-SA	PanAsia	O/BHU/22/2003	Eastern Region, Bhutan	Aug 16, 2003	Bovine	DQ165039
ME-SA	PanAsia	O/BHU/24/2003	Southern Region, Bhutan	Aug 16, 2003	Bovine	DQ165040
ME-SA	PanAsia	O/BHU/25/2003	Southern Region, Bhutan	Aug 16, 2003	Bovine	DQ164866†
ME-SA	PanAsia	O/BHU/41/2003	West Central Region, Bhutan	Oct 20, 2003	Bovine	DQ165041
ME-SA	PanAsia	O/BHU/47/2003	Southern Region, Bhutan	Dec 10, 2003	Bovine	DQ165042
ME-SA	PanAsia	O/BHU/49/2003	Western Region, Bhutan	Dec 31, 2003	Bovine	DQ164867†
ME-SA	PanAsia	O/BHU/26/2004	Western Region, Bhutan	Mar 2004	Bovine	DQ165043
ME-SA	PanAsia	O/BHU/27/2004	Western Region, Bhutan	Mar 2004	Bovine	DQ164868†
ME-SA	PanAsia	O/BHU/28/2004	Western Region, Bhutan	Mar 2004	Bovine	DQ165044
ME-SA	PanAsia	O/BHU/30/2004	Bhutan	Feb 16, 2004	Bovine	DQ165045
ME-SA	PanAsia	O/BHU/31/2004	Bhutan	Feb 16, 2004	Bovine	DQ164869†
ME-SA	PanAsia	O/BHU/33/2004	Bhutan	Feb 27, 2004	Bovine	DQ165046
ME-SA	PanAsia	O/BHU/39/2004	Bhutan	Apr 7, 2004	Bovine	DQ164870†
ME-SA	PanAsia	O/BHU/40/2004	Bhutan	Apr 7, 2004	Bovine	DQ165047
SEA	-	O/CAM/3/98	Kg. Speu, Cambodia	May 1, 1998	Bovine	AJ294910
ME-SA	PanAsia	O/CAM/2/2000	Angkor Chum, Siem Reap, Cambodia	Jan 27, 2000	Bovine	AJ318828†
ME-SA	PanAsia	O/CAM/4/2000	Angkor Chum, Siem Reap, Cambodia	Jan 29, 2000	Bovine	AJ318829†
ME-SA	PanAsia	O/Tibet/CHA/99	Tibet, P.R. China	1999	Bovine	AJ539138
WA	-	O/CIV/8/99	Côte d’Ivoire	1999	Bovine	AJ303485
ME-SA	-	O1/Sharquia/EGY/72	Sharquia Governate, Egypt	1972	Bovine	DQ164871†
Euro-SA	-	O1/Kaufbeuren/FRG/66	Kaufbeuren, Germany	1966	Bovine	X00871
ME-SA	PanAsia	O/FRA/1/2001	Mayenne, Pays de la Loire, France	Mar 2001	Not known	DQ164872†
ME-SA	PanAsia	O/FRA/1/2001	Mayenne, Pays de la Loire, France	Mar 2001	Not known	AJ633821
WA	-	O/GHA/5/93	Kintampo, Ghana	Jan 6, 1993	Bovine	AJ303488
WA	-	O/GNA/4/99	Kawkan/Madina, Guinea	May 8, 1999	Bovine	DQ165071
ME-SA	Branch A	O/GRE/23/94	Xanthi, Greece	Aug 4, 1994	Bovine	DQ164873†
ME-SA	Iran2001	O/GRE/18/96	Issakion, Didymotiho, Evros, Greece	Jul 31, 1996	ovine	DQ164874†
Cathay	-	O/HKN/21/70	Hang Tau, Sheung Shiu, N. T., Kowloon, Hong Kong	Mar 13, 1970	Porcine	AJ294911
Cathay	-	O/HKN/6/83	Pokfulam, Hong Kong Island	Dec 18, 1982	Bovine	AJ294919
Cathay	-	O/HKN/12/91	Sek Kwu Chau, N.T., Kowloon, Hong Kong	Nov 26, 1991	Porcine	AJ294921
Cathay	-	O/HKN/16/96	Takwuling, N.T., Kowloon, Hong Kong	Mar 29, 1996	Porcine	AJ294923
Cathay	-	O/HKN/20/96	Hong Kong	Apr 17, 1996	Bovine	AJ294924
Cathay	-	O/HKN/1/99	Mong Tseng Tsuen, Yuen Long, Hong Kong	Jan 5, 1998	Porcine	AJ294925
Cathay	-	O/HKN/4/2001	Hong Kong	2001	Not known	DQ164875†
Cathay	-	O/HKN/19/2001	Sheun Shui, NT, Hong Kong	Sep 28, 2001	Porcine	DQ164876†
Cathay	-	O/HKN/1/2002	Yuen Long, New Territories, Hong Kong	Jan 22, 2002	Porcine	DQ164877†
Cathay	-	O/HKN/3/2002	Yuen Long, New Territories, Hong Kong	Jan 31, 2002	Porcine	DQ164878†
Cathay	-	O/HKN/2/2003	Hong Kong	2003	Porcine	DQ164879†
Cathay	-	O/HKN/3/2003	Hong Kong	2003	Porcine	DQ164880†
Cathay	-	O/HKN/3/2004	Hong Kong	Jan 28, 2004	Porcine	DQ164881†
Cathay	-	O/HKN/4/2004	Hong Kong	Feb 11, 2004	Porcine	DQ164882†
Cathay	-	O/HKN/6/2004	Hong Kong	Mar 2, 2004	Porcine	DQ164883†
Cathay	-	O/HKN/7/2004	Hong Kong	Mar 18, 2004	Porcine	DQ164884†
Cathay	-	O/HKN/8/2004	Hong Kong	Aug 11, 2004	Porcine	DQ164885†
Cathay	-	O/HKN/9/2004	Hong Kong	Aug 11, 2004	Porcine	DQ164886†
Cathay	-	O/HKN/10/2004	Hong Kong	Aug 11, 2004	Porcine	DQ164887†
Cathay	-	O/HKN/11/2004	Hong Kong	Aug 11, 2004	Porcine	DQ164888†
Cathay	-	O/HKN/12/2004	Hong Kong	Aug 11, 2004	Porcine	DQ164889†
ME-SA	-	O5/IND/1/62	Thakurdwara, Moradabad, Uttar Pradesh, India	1962	Not known	DQ164890†
ME-SA	Branch B	O5/IND/62	Thakurdwara, Moradabad, Uttar Pradesh, India	1962	Not known	AY593828
ME-SA	Branch B	O5/IND/1/62	Thakurdwara, Moradabad, Uttar Pradesh, India	1962	Not known	Ref. 16
ME-SA	Branch B	O/IND/R2/75*	Tamilnadu, India	1975	Bovine	AF204276
ME-SA	Branch A	O/IND/53/79	Tamilnadu, India	1979	Bovine	AF292107
ME-SA	Branch A	O/IND/136/87*	Guwahati, Assam, India	1987	Bovine	Ref. 16
ME-SA	Branch A	O/IND/4/88*	Bareilly, Uttar Pradesh, India	1988	Bovine	Ref. 16
ME-SA	PanAsia	O/IND/231/88*	Kheda, Gujarat, India	1982	Bovine	Ref. 16
ME-SA	Branch A	O/IND/8/89*	Bangalore, Karnataka, India	1989	Bovine	Ref. 16
ME-SA	PanAsia	O/IND/6/90*	Jallandhar, Punjab, India	1990	Bovine	Ref. 16
ME-SA	Branch B	O/IND/79/90*	Jind, Haryana, India	1988	Bovine	Ref. 16
ME-SA	Branch A	O/IND/100/90*	Jallandhar, Punjab, India	1989	Porcine	Ref. 16
ME-SA	PanAsia	O/IND/232/90*	Cuttack, Orissa, India	1990	Bovine	Ref. 16
ME-SA	PanAsia	O/IND/234/90*	Cuttack, Orissa, India	1990	Bovine	Ref. 16
ME-SA	PanAsia	O/IND/235/90*	Cuttack, Orissa, India	1990	Bovine	Ref. 16
ME-SA	Branch A	O/IND/7/94*	Bangalore, Karnataka, India	1993	Bovine	Ref. 16
ME-SA	Iran2001	O/IND/27/95*	Salem, Tamilnadu, India	1994	Bovine	Ref. 16
ME-SA	Iran2001	O/IND/52/95*	Bhiwani, Haryana, India	1995	Bovine	Ref. 16
ME-SA	PanAsia	O/IND/50/96*	Thane, Maharastra, India	1990	Bovine	Ref. 16
ME-SA	Iran2001	O/IND/162/97*	Jalgaon, Maharastra, India	1997	Bovine	Ref. 16
ME-SA	Branch C-I	O/IND/279/97*	Nagaon, Assam, India	1997	Bovine	Ref. 16
ME-SA	PanAsia	O/IND/289/97*	Howrah, West Bengal, India	1997	Bovine	Ref. 16
ME-SA	Ind2001	O/IND/313/97*	Hisar, Haryana, India	1997	Bovine	Ref. 16
ME-SA	Branch A	O/IND/489/97*	Tamilnadu, India	1984	Not known	Ref. 16
ME-SA	PanAsia	O/IND/31/98*	Jalpaiguri, West Bengal, India	1997	Bovine	Ref. 16
ME-SA	PanAsia	O/IND/146/98*	Tumkur, Karnataka, India	1998	Porcine	Ref. 16
ME-SA	Branch C-I	O/IND/413/98*	Doddaballapura, Karnataka, India	1998	Bovine	Ref. 16
ME-SA	PanAsia	O/IND/429/98*	Anantpur, Andhra Pradesh, India	1998	Bovine	Ref. 16
ME-SA	Branch C-I	O/IND/141/99*	Thirunelveli, Tamilnadu, India	1999	Buffalo	Ref. 16
ME-SA	PanAsia	O/IND/143/99*	Agra, Uttar Pradesh, India	1999	Bovine	Ref. 16
ME-SA	PanAsia	O/IND/209/99*	Ahmednagar, Maharashtra, India	1998	Bovine	Ref. 16
ME-SA	Ind2001	O/IND/246/99*	Gujarat, India	1999	Bovine	Ref. 16
ME-SA	PanAsia	O/IND/5/00*	Kurnool, Andhra Pradesh, India	1999	Bovine	Ref. 16
ME-SA	PanAsia	O/IND/30/00*	Sirsa, Haryana, India	1999	Bovine	Ref. 16
ME-SA	PanAsia	O/IND/34/00*	Hisar, Haryana, India	2000	Bovine	Ref. 16
ME-SA	PanAsia	O/IND/36/00*	Sirsa, Haryana, India	2000	Bovine	Ref. 16
ME-SA	PanAsia	O/IND/43/00*	Chitradurga, Karnataka, India	1999	Bovine	Ref. 16
ME-SA	PanAsia	O/IND/45/00*	Doddaballapura, Karnataka, India	1999	Bovine	Ref. 16
ME-SA	PanAsia	O/IND/74/00*	Hassan, Karnataka, India	2000	Bovine	Ref. 16
ME-SA	PanAsia	O/IND/75/00*	Arakalgudu, Karnataka, India	2000	Bovine	Ref. 16
ME-SA	PanAsia	O/IND/77/00*	Arakalgudu, Karnataka, India	2000	Bovine	Ref. 16
ME-SA	Iran2001	O/IND/90/00*	Chitradurga, Karnataka, India	2000	Bovine	Ref. 16
ME-SA	PanAsia	O/IND/91/00*	Chitradurga, Karnataka, India	2000	Bovine	Ref. 16
ME-SA	Iran2001	O/IND/111/00*	Howrah, West Bengal, India	1999	Bovine	Ref. 16
ME-SA	PanAsia	O/IND/112/00*	Nadia, West Bengal, India	2000	Bovine	Ref. 16
ME-SA	Iran2001	O/IND/151/00*	Midnapore, West Bengal, India	2000	Bovine	Ref. 16
ME-SA	Iran2001	O/IND/156/00*	Nadia, West Bengal, India	2000	Bovine	Ref. 16
ME-SA	Iran2001	O/IND/157/00*	24 Parganas, West Bengal, India	2000	Bovine	Ref. 16
ME-SA	Iran2001	O/IND/398/00*	Surendra Nagar, Gujarat, India	2000	Not known	Ref. 16
ME-SA	Ind2001	O/IND/402/00*	Jam Nagar, Gujarat, India	2000	Not known	Ref. 16
ME-SA	Iran2001	O/IND/40/01*	Midnapore, West Bengal, India	2000	Bovine	Ref. 16
ME-SA	Ind2001	O/IND/58/01*	Mahadevpur, Karnataka, India	2001	Bovine	Ref. 16
ME-SA	Ind2001	O/IND/64/01*	Muzaffarnagar, Uttar Pradesh, India	2001	Bovine	Ref. 16
ME-SA	PanAsia	O/IND/78/01*	Hoshiarpur, Punjab, India	2001	Bovine	Ref. 16
ME-SA	PanAsia	O/IND/79/01*	Hoshiarpur, Punjab, India	2001	Bovine	Ref. 16
ME-SA	Ind2001	O/IND/83/01*	Mandya, Karnataka, India	2001	Bovine	Ref. 16
ME-SA	Ind2001	O/IND/86/01*	Kolar, Karnataka, India	2001	Bovine	Ref. 16
ME-SA	Ind2001	O/IND/93/01*	Kolar, Karnataka, India	2001	Bovine	Ref. 16
ME-SA	Ind2001	O/IND/96/01*	Ludhiana, Punjab, India	2001	Buffalo	Ref. 16
ME-SA	Ind2001	O/IND/97/01*	Ambala, Haryana, India	2001	Bovine	Ref. 16
ME-SA	PanAsia	O/IND/102/01*	Hatharas, Uttar Pradesh, India	2001	Buffalo	Ref. 16
ME-SA	Ind2001	O/IND/106/01*	Hisar, Haryana, India	2001	Bovine	Ref. 16
ME-SA	Ind2001	O/IND/111/01*	Gurgaon, Haryana, India	2001	Bovine	Ref. 16
ME-SA	Ind2001	O/IND/112/01*	Gurgaon, Haryana, India	2001	buffalo	Ref. 16
ME-SA	PanAsia	O/IND/115/01*	Kuruksetra, Haryana, India	2001	Bovine	Ref. 16
ME-SA	Ind2001	O/IND/116/01*	Narnaul, Haryana, India	2001	Bovine	Ref. 16
ME-SA	Ind2001	O/IND/118/01*	Jaipur, Rajasthan, India	2001	buffalo	Ref. 16
ME-SA	Ind2001	O/IND/119/01*	Karnal, Haryana, India	2001	Bovine	Ref. 16
ME-SA	Ind2001	O/IND/125/01*	Faridabad, Haryana, India	2001	Bovine	Ref. 16
ME-SA	PanAsia	O/IND/136/01*	Baruch, Gujarat, India	2001	Bovine	Ref. 16
ME-SA	PanAsia	O/IND/151/01*	Kutch, Gujarat, India	2001	Bovine	Ref. 16
ME-SA	PanAsia	O/IND/155/01*	Baruch, Gujarat, India	2001	Bovine	Ref. 16
ISA-1	-	O/ISA/1/62	Bali, Indonesia	Jul 1962	Bovine	AJ303500
ISA-2	-	O/JAV/5/72	Java, Indonesia	1972	Not known	AJ303509
ISA-2	-	O/ISA/1/74	Bali Quarantine Station, Indonesia	1974	Bovine	AJ303501
ISA-1	-	O/ISA/9/74	Bali, Indonesia	Oct 30, 1974	Bovine	AJ303502
ISA-1	-	O/ISA/8/83	East Java, Indonesia	1983	Bovine	AJ303503
ME-SA	PanAsia	O/IRL/134/2001	Louth, Republic of Ireland	Mar 20, 2001	Bovine	DQ164891†
ME-SA	PanAsia	O/IRN/9/99	Iran	1999	Not known	AJ318838†
ME-SA	PanAsia	O/IRN/24/99	Iran	1999	Not known	AJ318839†
ME-SA	PanAsia	O/IRN/1/2000	Ghotil, West Azrarbijan, Mahabad, Iran	May 6, 2000	Bovine	DQ164892†
ME-SA	PanAsia	O/IRN/16/2000	Sardasht, W. Azerbaijan, Iran	Jun 28, 2000	Bovine	AJ318840†
ME-SA	PanAsia	O/IRN/16/2001	Shirin Abad, Markazy, Iran	Jul 9, 2001	Bovine	DQ164893†
ME-SA	PanAsia	O/IRN/41/2001	Oshnaveye, West Azar, Iran	Nov 14, 2001	Bovine	DQ164894†
ME-SA	PanAsia	O/IRN/58/2001	Dam Shahz, Qom, Iran	Nov 29, 2001	Bovine	DQ164895†
ME-SA	Iran2001	O/IRN/61/2001	Mynab, Hormozgan, Iran	Dec 15, 2001	Bovine	DQ164896†
ME-SA	PanAsia	O/IRN/67/2001	Zahedan, Systan, Iran	Dec 22, 2001	Bovine	DQ164897†
ME-SA	PanAsia	O/IRN/2/2003	Abaz Abad, West Azarbijan, Iran	Jan 3, 2003	Bovine	DQ165048
ME-SA	Iran2001	O/IRN/4/2003	Kahak, Qom, Iran	Jan 4, 2003	Bovine	DQ165049
ME-SA	Iran2001	O/IRN/6/2003	Hajiabad, Hormozgan, Iran	Jan 9, 2003	Bovine	DQ165050
ME-SA	Iran2001	O/IRN/8/2003	Sanandoj, Kordestan, Iran	Jan 15, 2003	Bovine	DQ165051
ME-SA	Iran2001	O/IRN/15/2003	Amol, Mazanderan, Iran	Apr 13, 2003	Not known	DQ164898†
ME-SA	PanAsia	O/IRN/16/2003	Gorgah, Golestan, Iran	Apr 14, 2003	Bovine	DQ165052
ME-SA	PanAsia	O/IRN/6/2004	Oshnavieh, W. Azerbaijan, Iran	Jul 11, 2004	Bovine	DQ165053
ME-SA	PanAsia	O/IRN/8/2004	Urmiah, W. Azarbaijan, Iran	Aug 17, 2004	Bovine	DQ165054
ME-SA	Iran2001	O/IRN/15/2004	Ardebil, Ardebil, Iran	Oct 3, 2004	Bovine	DQ165055
ME-SA	Iran2001	O/IRN/20/2004	Hosseinabad, Fars, Shiraz, Iran	Oct 19, 2004	Bovine	DQ165056
ME-SA	PanAsia	O/IRQ/26/2000	Erbil, Iraq	Apr 9, 2000	Bovine	AJ318841†
ME-SA	PanAsia	O/IRQ/30/2000	Erbil, Iraq	Apr 9, 2000	Bovine	DQ165057
ME-SA	-	O/Geshur/ISR/85	Kibbutz Geshur, Galand, Israel	May 1985	Bovine	AF189157
ME-SA	-	O/ISR/2/88	Dalton, Zefat, Israel	Jun 24, 1988	Bovine	DQ164899†
ME-SA	PanAsia	O/ISR/2/2004	Hadaron, Israel	Jan 20, 2004	Bovine	DQ164900†
ME-SA	PanAsia	O/ISR/3/2004	Hadaron, Israel	Jan 22, 2004	Bovine	DQ164901†
ME-SA	PanAsia	O/ISR/4/2004	Hazafon, Israel	Jan 23, 2004	Bovine	DQ164902†
Euro-SA	-	O1/Lombardy/ITL/46	Lombardy, Italy	1946	Not known	M58601
Euro-SA	-	O2/Brescia/ITL/47	Brescia, Italy	1947	Not known	M55287
ME-SA	PanAsia	O/JPN/2000	Miyazaki, Japan	Apr 7, 2000	Bovine	AB079061
ME-SA	Iran2001	O/JOR/2/95	Amman Governorate, Jordan	Oct 30, 1995	Bovine	DQ164903†
EA-1	-	O/K77/78*	Manera Est., Naivasha, Kenya	1978	Not known	DQ165072
EA-1	-	O/K83/79*	Mweiga, Nyeri Dist., Central Prov., Kenya	1979	Bovine	AJ303511
EA-2		O/KEN/5/2002	Nakuru, Lanet Division, Kenya	Oct 9, 2002	Bovine	DQ165073
ME-SA	Ind2001	O/KUW/3/97	Kuwait	1997	Bovine	DQ164904†
ME-SA	PanAsia	O/KUW/1/98	Warfra (Al Warfah), Kuwait	May 2, 1998	Bovine	DQ164905†
SEA	-	O/LAO/4/98	Attapeu, Laos	Sep 24, 1998	Buffalo	DQ164906†
ME-SA	PanAsia	O/LAO/2/2000	Kephathao, Sayabluly, Laos	Jan 26, 2000	Not known	AJ318844†
SEA	-	O/LAO/4/2001	Xieng Khouang, Laos	Aug 3, 2001	Bovine	DQ164907†
ME-SA	PanAsia	O/LAO/3/2003	Xaythany, VT Municipality, Laos	Mar 21, 2003	Bovine	DQ164908†
ME-SA	PanAsia	O/LAO/11/2003	Xaysettha, VT Municipality, Laos	May 12, 2003	Buffalo	DQ164909†
ME-SA	PanAsia	O/LAO/12/2003	Hatxaifong, VT Municipality, Laos	May 15, 2003	Bovine	DQ164910†
ME-SA	PanAsia	O/LAO/13/2003	Xaysettha, VT Municipality, Laos	May 16, 2003	Buffalo	DQ164911†
ME-SA	PanAsia	O/LAO/17/2003	Tonepheung, Borkeo, Laos	May 25, 2003	Buffalo	DQ164912†
ME-SA	PanAsia	O/LAO/19/2003	Tonepheung, Borkeo, Laos	May 27, 2003	Bovine	DQ164913†
ME-SA	PanAsia	O/LAO/21/2003	Chanthabuly, VT Municipality, Laos	Jun 25, 2003	Porcine	DQ164914†
ME-SA	PanAsia	O/LAO/23/2003	Chanthabuly, VT Municipality, Laos	Jun 26, 2003	Porcine	DQ164915†
ME-SA	PanAsia	O/LAO/28/2003	Thaphabath, Borikhamxai, Laos	Sep 16, 2003	Bovine	DQ164916†
ME-SA	PanAsia	O/LAO/30/2003	Thaphabath, Borikhamxai, Laos	Oct 4, 2003	Porcine	DQ164917†
ME-SA	PanAsia	O/LAO/31/2003	Thaphabath, Borikhamxai, Laos	Oct 4, 2003	Porcine	DQ164918†
ME-SA	PanAsia	O/LEB/1/2003	Lebanon	Mar 26, 2003	Bovine	DQ164919†
EA-2	-	O/MAL/1/98	Mwongulukulu dip tank, Karonga, Northern Malawi	Nov 6, 1998	Not known	DQ165074
ME-SA	PanAsia	O/MAY/2/2000	Kilang Papan, Batu Arang, Selangor, Malaysia	Jan 2, 2000	Bovine	AJ318846†
SEA	-	O/MAY/3/2001	S.G Kelambu Kuala Langat, Selangor, Malaysia	Jun 23, 2001	Bovine	DQ164920†
SEA	-	O/MAY/5/2001	Twluk Jering, Kelantan, Tumpat, Malaysia	Jul 11, 2001	Bovine	DQ164921†
SEA	-	O/MAY/6/2001	Twluk Jering, Kelantan, Tumpat, Malaysia	Jul 11, 2001	Bovine	DQ164922†
SEA	-	O/MAY/1/2002	Pengkala Huln, Perak, Malaysia	Jan 10, 2002	Bovine	DQ164923†
ME-SA	PanAsia	O/MAY/6/2003	Rompin, Pahang, Malaysia	Dec 28, 2003	Bovine	DQ165058
SEA	-	O/MYA/13/89	Kyaik Latt, Myanmar	Jul 20, 1989	Bovine	DQ164924†
SEA	-	O/MYA/7/98	U Uin Myint, Warr Ta Ya, Htan Ta Pin, Yangon, Myanmar	Jul 14, 1998	Bovine	DQ164925†
SEA	-	O/MYA/5/99	U Khin Win, Tha Pyay Sein, Zigon, Bago, Myanmar	Jun 8, 1999	Bovine	DQ164926†
SEA	-	O/MYA/2/2000	Kant Malar, Yangon, Kungyaw Kone, Myanmar	May 23, 2000	Bovine	DQ164927†
SEA	-	O/MYA/7/2002	Hlayhlaninn, Hlegu, Yangon, Myanmar	Oct 23, 2002	Porcine	DQ164928†
ME-SA	PanAsia	O/NEP/111/90	Kathmandu, Nepal	May 17, 1990	Porcine	DQ164929†
ME-SA	PanAsia	O/NEP/121/90	Bhojpur, Nepal	Sep 23, 1990	Bovine	DQ164930†
ME-SA		O/NEP/6/91	Kathmandu, Nepal	Jun 5, 1991	Bovine	DQ164931†
ME-SA		O/NEP/4/92	Kathmandu, Nepal	Oct 22, 1992	Bovine	DQ164932†
ME-SA	PanAsia	O/NEP/104/97	k.b. Budhathoki, Kathmandu, Nepal	Oct 16, 1997	Bovine	DQ164933†
ME-SA	PanAsia	O/NEP/109/97	Okhaldhunga, Nepal	Nov 30, 1997	buffalo	DQ164934†
ME-SA	PanAsia	O/NEP/4/98	Kathmandu, Nepal	Apr 30, 1998	Porcine	DQ164935†
ME-SA	PanAsia	O/NEP/9/98	Lalitpur, Nepal	Oct 4, 1998	Bovine	DQ164936†
ME-SA	PanAsia	O/NEP/2/99	Pyuthan, Nepal	Jan 4, 1999	Bovine	DQ164937†
ME-SA	-	O/NEP/12/2000	Kathmandu, Nepal	Jun 14, 2000	Bovine	DQ164938†
ME-SA	-	O/NEP/13/2000	Kathmandu, Nepal	Jun 14, 2000	Bovine	DQ164939†
ME-SA	PanAsia	O/NEP/4/2003	Lalitpur, Nepal	May 9, 2003	Bovine	DQ165059
ME-SA	PanAsia	O/NEP/5/2003	Gagalphed 1-7, Kathmandu, Nepal	May 29, 2003	Bovine	DQ165060
ME-SA	PanAsia	O/NEP/6/2003	Gagalphed 1-7, Kathmandu, Nepal	Jun 9, 2003	Bovine	DQ165061
ME-SA	Ind2001	O/OMN/4/2001	Oman	2001	Caprine	DQ164940†
ME-SA	Ind2001	O/OMN/7/2001	Oman	2001	Bovine	DQ164941†
ME-SA	Iran2001	O/PAK/15/2002	Sheikh Pura, Punjab, Pakistan	Mar 15, 2002	Buffalo	DQ165062
ME-SA	Iran2001	O/PAK/16/2002	Pakistan	Mar 15, 2002	Bovine	DQ165063
ME-SA	Iran2001	O/PAK/18/2002	Karachi, Pakistan	Jul 27, 2002	Bovine	DQ165064
ME-SA	Iran2001	O/PAK/1/2003	Karachi, Pakistan	Jan 6, 2003	Bovine	DQ165065
ME-SA	Pak98	O/PAK/12/2003	Lahore, Punjab, Pakistan	2003	Not known	DQ165066
ME-SA	Pak98	O/PAK/14/2003	Lahore, Punjab, Pakistan	2003	Not known	DQ165067
ME-SA	Pak98	O/PAK/16/2003	Lahore, Punjab, Pakistan	2003	Not known	DQ165068
ME-SA	Pak98	O/PAK/17/2003	Lahore, Punjab, Pakistan	2003	Not known	DQ165069
ME-SA	PanAsia	O/PAK/45/2003	Pakistan	May 19, 2003	Bovine	DQ164942†
ME-SA	Iran2001	O/PAK/53/2003	Pakistan	May 27, 2003	Buffalo	DQ164943†
ME-SA	Pak98	O/PAK/73/2003	Pakistan	Oct 27, 2003	Not known	DQ165070
ME-SA	Ind2001	O/PAT/2/2002	Deir Dibwan, Ramallah, Palestinian Autonomous Territories	2002	Bovine	DQ164944†
ME-SA	Ind2001	O/PAT/3/2002	Nr. Ramallah, Palestinian Autonomous Territories	Nov 12, 2002	Bovine	DQ164945†
Cathay	-	O/PHI/5/95	Bauang, La Union, Philippines	Feb 9, 1995	Porcine	DQ164946†
Cathay	-	O/PHI/7/96	Mahabang Parang, Angono, Rizal, Philippines	Nov 1, 1996	Porcine	AJ294926
Cathay	-	O/PHI/5/2000	(Cabanatuan), 111, Nueva Ecija, Philippines	Feb 8, 2000	Porcine	DQ164947†
Cathay	-	O/PHI/13/2000	Staana, 111, Pamapanga, Philippines	Mar 2, 2000	Porcine	DQ164948†
Cathay	-	O/PHI/14/2000	Vicotria, 1V, Oriental Mindoro, Philippines	Mar 11, 2000	Porcine	DQ164949†
Cathay	-	O/PHI/5/2003	Bangued, CAR, Abra, Philippines	Feb 10, 2003	Porcine	DQ164950†
Cathay	-	O/PHI/10/2003	Muntinlupa City, NCR, Muntinlupa, Philippines	Mar 4, 2003	Porcine	DQ164951†
Cathay	-	O/PHI/17/2003	Guagua, 111, Pampanga, Philippines	Apr 8, 2003	Porcine	DQ164952†
Cathay	-	O/PHI/21/2003	Antipolo City, 1V, Rizal, Philippines	May 13, 2003	Porcine	DQ164953†
Cathay	-	O/PHI/23/2003	Cainta, 1V, Rizal, Philippines	May 15, 2003	Porcine	DQ164954†
Cathay	-	O/PHI/1/2004	Cabaroandaya, 1, Vigan Ilocos Sur, Philippines	Jan 13, 2004	Porcine	DQ164955†
Cathay	-	O/PHI/2/2004	Guagua, II, Pampanga, Philippines	Jan 16, 2004	Porcine	DQ164956†
Cathay	-	O/PHI/3/2004	Quezon City, NCR, Metro Manila, Philippines	Jan 5, 2004	Porcine	DQ164957†
Cathay	-	O/PHI/4/2004	Tondo, NCR, Metro Manila, Philippines	Mar 24, 2004	Porcine	DQ164958†
Cathay	-	O/PHI/5/2004	Pinagbuhatan, NCR, Pasig City, Philippines	Jun 1, 2004	Porcine	DQ164959†
Cathay	-	O/PHI/6/2004	Caloocan City, NCR, Metro Manila, Philippines	Jun 21, 2004	Porcine	DQ164960†
Cathay	-	O/PHI/7/2004	Gerona, III, Tarlac, Philippines	Jun 29, 2004	Porcine	DQ164961†
Cathay	-	O/PHI/8/2004	Guiguinto, III, Bulacan, Philippines	Jul 14, 2004	Porcine	DQ164962†
Cathay	-	O/PHI/9/2004	Mayamot, Antipolo, IV B, Rizal, Philippines	Jul 21, 2004	Porcine	DQ164963†
Cathay	-	O/PHI/10/2004	NCR, Quezon City, Philippines	Aug 4, 2004	Porcine	DQ164964†
Cathay	-	O/PHI/11/2004	Muntinlupa, NCR, Metro Manila, Philippines	Sep 3, 2004	Porcine	DQ164965†
Cathay	-	O/PHI/12/2004	Binan, IV B, Laguna, Philippines	Sep 29, 2004	Porcine	DQ164966†
ME-SA	PanAsia	O/QTR/3/99	Al-Wabra, Wildlife Preservation, Dohar, Qatar	Feb 27, 1999	Dorcas gazelle	DQ164967†
ME-SA	PanAsia	O/SAR/1/2000	Camperdown, Kwazulu-Natal, South Africa	Sep 2000	Porcine	AJ318860†
ME-SA	-	O/SAU/2/97	Riyadh, Saudi Arabia	May 1997	Bovine	AJ318851†
ME-SA	PanAsia	O/SAU/38/98	Al‑Kharj, Saudi Arabia	1998	Bovine	AJ318852†
ME-SA	PanAsia	O/SAU/2/99	Saudi Arabia	1999	Not known	DQ164968†
ME-SA	Ind2001	O/SAU/3/2001	Asier, Saudi Arabia	Mar 2001	Bovine	DQ164969†
ME-SA	Ind2001	O/SAU/11/2001	Al Karj, Saudi Arabia	Jan 2001	Bovine	DQ164970†
ME-SA	PanAsia	O/SAU/13/2001	Almaria, Riyadh, Saudi Arabia	Oct 12, 2001	Bovine	DQ164971†
ME-SA	PanAsia	O/SKR/1/2000	Papyung, P’aju City, Kyunggi, Republic of Korea	Mar 26, 000	Bovine	AJ318854†
ME-SA	PanAsia	O/SKR/2000	ChungJu county, Kyunggi province, Republic of Korea	May 2000	Bovine	AJ539139
ME-SA	PanAsia	O/SKR/2000	Republic of Korea	2000	Not known	AF377945
ME-SA	PanAsia	O/SKR/2000	Republic of Korea	2000	Not known	AF428246
ME-SA	PanAsia	O/SKR/1/2002	Samjuk, Anseong, Kyonggi province, Republic of Korea	May 2, 2002	Porcine	DQ164972†
ME-SA	PanAsia	O/SKR/AS/2002	Samjuk, Anseong, Kyonggi province, Republic of Korea	2002	Porcine	AY114146
EA-3	-	O/SUD/2/86	Omdurman, Khartoum, Sudan	Nov 17, 1986	Bovine	DQ165075
EA-3	-	O/SUD/1/99	Sudan	1999	Not known	DQ165076
ME-SA	PanAsia	O/SYR/1/2002	Syria	Feb 2002	Not known	DQ164973†
ME-SA	PanAsia	O/SYR/2/2002	Syria	Feb 2002	Not known	DQ164974†
ME-SA	PanAsia	O/SYR/3/2002	Syria	Feb 2002	Not known	DQ164975†
SEA	-	O/TAI/4/99	Mae Hong Son, Thailand	Mar 1999	Bovine	DQ164976†
SEA	-	O/TAI/8/99	Buriram, Thailand	Jun 1999	Bovine	DQ164977†
ME-SA	PanAsia	O/TAI/9/99	Chieng Rai, Thailand	Jun 1999	Bovine	DQ164978†
SEA	-	O/TAI/2/2000	Songkhla, Thailand	Jan 18, 2000	Bovine	DQ164979†
ME-SA	PanAsia	O/TAI/2/2003	Nakhon Ratchasima, Thailand	Mar 10, 2003	Bovine	DQ164980†
SEA	-	O/TAI/3/2003	Nakompathom, Thailand	May 19, 2003	Porcine	DQ164981†
Cathay	-	O/TAW/81/97	I-lan, Taiwan POC	17/04/1997	Porcine	AJ296321
ME-SA	PanAsia	O/TAW/2/99	Kinmen, Taiwan POC	Jun 1999	Bovine	AJ294927
Cathay	-	O/TAW/4/99	Penghu Island, Taiwan POC	Feb 1999	Porcine	AJ294928
ME-SA	Branch B	O1/Manisa/TUR/69	Manisa, Turkey	Apr 1969	Bovine	AJ251477
ME-SA	Iran2001	O/TUR/2/2000	Merlez, Balikesir, Turkey	2000	Bovine	DQ164982†
ME-SA	PanAsia	O/TUR/5/2000	Nigde, Turkey	2000	Bovine	DQ164983†
ME-SA	PanAsia	O/TUR/8/2000	Konya, Turkey	2000	Bovine	DQ164984†
ME-SA	PanAsia	O/TUR/2/2001	Kastamonu, Kastamonu, Turkey	Jan 26, 2001	Bovine	DQ164985†
ME-SA	PanAsia	O/TUR/3/2002	Diyarbakir, Diyarbakir, Turkey	2002	Bovine	DQ164986†
ME-SA	Iran2001	O/TUR/5/2002	Sivas, Sivas, Turkey	2002	Bovine	DQ164987†
ME-SA	PanAsia	O/TUR/12/2002	Dozansehir, Malatya, Turkey	May 2002	Bovine	DQ164988†
ME-SA	PanAsia	O/TUR/1/2003	Aksaray, Turkey	2003	Bovine	DQ164989†
ME-SA	PanAsia	O/TUR/3/2003	Amasya, Turkey	2003	Bovine	DQ164990†
ME-SA	PanAsia	O/TUR/7/2003	Nigde, Turkey	2003	Bovine	DQ164991†
ME-SA	Ind2001	O/UAE/7/97	Al Ain, United Arab Emirates	May 1997	Bovine	DQ164992†
ME-SA	PanAsia	O/UAE/4/99	United Arab Emirates	1999	Antelope	DQ164993†
ME-SA	PanAsia	O/UAE/1/2000	Al Rawabi, Dubai, United Arab Emirates	May 6, 2000	Bovine	DQ164994†
ME-SA	PanAsia	O/UAE/2/2000	Al Rawabi, Dubai, United Arab Emirates	May 6, 2000	Bovine	DQ164995†
ME-SA	PanAsia	O/UAE/3/2000	Al Rawabi, Dubai, United Arab Emirates	May 6, 2000	Bovine	DQ164996†
ME-SA	Ind2001	O/UAE/6/2001	Al Ain, United Arab Emirates	Mar 2001	Bovine	DQ164997†
EA-2	-	O/UGA/3/2002	Nakasongola, Uganda	2002	Not known	DQ165077
ME-SA	PanAsia	O/UKG/12/2001	Brentwood, Essex, UK.	Feb 19, 2001	Porcine	AJ311724
ME-SA	PanAsia	O/UKG/31/2001	Brentwood, Essex, UK.	Feb 20, 2001	Bovine	DQ164998†
ME-SA	PanAsia	O/UKG/35/2001	Brentwood, Essex, UK.	Feb 20, 2001	Porcine	AJ539141
ME-SA	PanAsia	O/UKG/123/2001	Brentwood, Essex, UK.	Feb 22, 2001	Bovine	DQ164999†
ME-SA	PanAsia	O/UKG/128/2001	Heddon-on-the-Wall, Northumberland, UK.	Feb 22, 2001	Porcine	DQ165000†
ME-SA	PanAsia	O/UKG/130/2001	Canewdon, Essex, UK.	Feb 22, 2001	Porcine	DQ165001†
ME-SA	PanAsia	O/UKG/150/2001	Ponteland, Northumberland, UK.	Feb 23, 2001	Bovine	DQ165002†
ME-SA	PanAsia	O/UKG/152/2001	Ponteland, Northumberland, UK.	Feb 23, 2001	Bovine	DQ165003†
ME-SA	PanAsia	O/UKG/174/2001	Beaworthy, Highamton, Devon, UK.	Feb 24, 2001	Bovine	DQ165004†
ME-SA	PanAsia	O/UKG/195/2001	Bromham, Chippenham, Wiltshire, UK.	Feb 2001	Bovine	DQ165005†
ME-SA	PanAsia	O/UKG/198/2001	Burdon, Hatherleigh, Okehampton, Devon, UK.	Feb 25, 2001	Bovine	DQ165006†
ME-SA	PanAsia	O/UKG/240/2001	Llangarron, Ross-on-Wye, Herefordshire, UK.	Feb 26, 2001	Bovine	DQ165007†
ME-SA	PanAsia	O/UKG/241/2001	Llangarron, Ross-on-Wye, Herefordshire, UK.	Feb 26, 2001	Bovine	DQ165008†
ME-SA	PanAsia	O/UKG/438/2001	County Armagh, Northern Ireland, UK.	Feb 28, 2001	Bovine	DQ165009†
ME-SA	PanAsia	O/UKG/478/2001	Dryfesdale, Lockerbie, Dumfries & Galloway, UK.	Feb 28, 2001	Bovine	DQ165010†
ME-SA	PanAsia	O/UKG/3730/2001	Bogues, Ecclefechan, Lockerbie, Dumfries & Galloway, UK.	Mar 30, 2001	Not known	DQ165011†
ME-SA	PanAsia	O/UKG/3802/2001	Milton Damerel, Holsworthy, Devon, UK.	Mar 30, 2001	Bovine	DQ165012†
ME-SA	PanAsia	O/UKG/4021/2001	Cockermouth, Cumbria, UK.	Apr 1, 2001	Bovine	DQ165013†
ME-SA	PanAsia	O/UKG/4027/2001	Hawes, North Yorkshire, UK.	Mar 29, 2001	Bovine	DQ165014†
ME-SA	PanAsia	O/UKG/4553/2001	Jedburgh, Borders, UK.	Apr 6, 2001	Bovine	DQ165015†
ME-SA	PanAsia	O/UKG/5060/2001	Coagh, County Tyrone, Northern Ireland, UK.	Apr 11, 2001	Bovine	DQ165016†
ME-SA	PanAsia	O/UKG/5565/2001	Cushendall, County Antrim, Northern Ireland, UK.	Apr 14, 2001	Bovine	DQ165017†
ME-SA	PanAsia	O/UKG/9359/2001	Crathorne, Yarm, North Yorkshire, UK.	May 16, 2001	Bovine	DQ165018†
ME-SA	PanAsia	O/UKG/13726/2001	Abram, Wigan, Lancashire, UK	Jul 16, 2001	Bovine	DQ165019†
Euro-SA	-	O3/VEN/51	Venezuela	1951	Not known	AJ004645
SEA	-	O/VIT/2/97	Vietnam	1997	Bovine	AJ294929
Cathay	-	O/VIT/3/97	Vietnam	1997	Porcine	AJ294930
SEA	-	O/VIT/7/97	Uar, Krong Pa, Gialai, Region no. 37, Vietnam	Aug 31, 1997	Not known	AJ296328
ME-SA	PanAsia	O/VIT/6/2002	Vietnam	2002	Not known	DQ165020†
ME-SA	PanAsia	O/VIT/8/2002	Vietnam	2002	Not known	DQ165021†
ME-SA	PanAsia	O/VIT/9/2002	Vietnam	2002	Not known	DQ165022†
ME-SA	PanAsia	O/VIT/10/2002	Vietnam	2002	Not known	DQ165023†
ME-SA	PanAsia	O/VIT/12/2002	Vietnam	2002	Not known	DQ165024†
Cathay	-	O/VIT/13/2002	Vietnam	2002	Not known	DQ165025†
ME-SA	PanAsia	O/VIT/14/2002	Kien Giang, Vietnam	Feb 2002	Porcine	DQ165026†
ME-SA	PanAsia	O/VIT/16/2002	Kien Giang, Vietnam	Mar 2002	Porcine	DQ165027†
ME-SA	PanAsia	O/VIT/19/2002	Kontum, Vietnam	Jun 2002	Bovine	DQ165028†
ME-SA	PanAsia	O/VIT/20/2002	Binh Thuan, Vietnam	Dec 2002	Bovine	DQ165029†
ME-SA	PanAsia	O/VIT/1/2003	Phu Yen, Vietnam	2003	Porcine	DQ165030†
ME-SA	PanAsia	O/VIT/2/2003	An Giang, Vietnam	Aug 2003	Porcine	DQ165031†
ME-SA	PanAsia	O/VIT/1/2004	Kon Tum, Vietnam	Feb 2004	Bovine	DQ165032†
Cathay	-	O/VIT/2/2004	Quang Nam, Vietnam	Feb 2004	Porcine	DQ165033†
Cathay	-	O/VIT/3/2004	Quang Nam, Vietnam	Mar 2004	Porcine	DQ165034†
*Not a reference number of the World Reference Laboratory for Foot-and-Mouth Disease. †Sequence determined in this study.						

**Table A2:** Occurrence of the PanAsia lineage of foot-and-mouth disease virus serotype O, 1990–2004*† Download PDF (45 KB, 3 pages)

Country	1990	1991	1992	1993	1994	1995	1996	1997	1998	1999	2000	2001	2002	2003	2004
India	ME-SA	ME-SA	ME-SA	ME-SA	ME-SA	ME-SA	ME-SA	ME-SA	ME-SA	ME-SA	ME-SA	ME-SA	+	+	+
Nepal		ME-SA	ME-SA	ME-SA	ME-SA	ME-SA	ME-SA				ME-SA			ME-SA	+
Bhutan	+	+		ME-SA	+	+				+	+		ME-SA		
Iran	+	+	+	ME-SA	ME-SA	ME-SA	+	ME-SA				+		ME-SA	ME-SA
Jordan	+			ME-SA	ME-SA	ME-SA	ME-SA								
Syria	+	ME-SA	+	-	ME-SA										
Saudi Arabia	ME-SA	ME-SA	ME-SA	ME-SA	ME-SA	ME-SA	?	ME-SA						-	
Yemen	+	+	+	+		ME-SA				+				EA-3	EA-3
Lebanon	+	+	ME-SA	ME-SA		ME-SA	?	?	ME-SA						
Kuwait	+	+	-		ME-SA	ME-SA	ME-SA	ME-SA						+	
Bahrain	+	ME-SA	ME-SA	ME-SA	+	ME-SA	ME-SA	ME-SA				+		+	-
Qatar			-	-								+			
Iraq					+										
UAE	+		ME-SA	+		-		ME-SA				ME-SA		ME-SA	-
Turkey	ME-SA	ME-SA	ME-SA	ME-SA	ME-SA	ME-SA	ME-SA	+	ME-SA		ME-SA		ME-SA		+
Israel	+	ME-SA	ME-SA	+	ME-SA	ME-SA	ME-SA	?	?					-	
P.R. China			+						+						
Taiwan POC	-	-	-	-	-	-	-	CHY	CHY	CHY	CHY				
Sri Lanka	+	ME-SA	+	+	+			ME-SA	+	ME-SA	+			+	
Myanmar	+	+		+	+	+	SEA		SEA	SEA			SEA	+	SEA
Thailand	+	SEA	SEA	+	SEA	SEA		+	+	SEA	SEA	+	SEA	SEA	+
Vietnam	-	-	-			+		SEA	+	SEA/CHY			CHY	CHY	CHY
Laos	+	+		SEA				-	SEA					SEA	
Malaysia	-	-	SEA	+	SEA	ME-SA	SEA	+	-	SEA		SEA	SEA	SEA	
Cambodia	+	-	SEA	+	SEA	+	+	+	SEA	SEA					
Japan	-	-	-	-	-	-	-	-	-	-		-	-	-	-
South Korea	-	-	-	-	-	-	-	-	-	-				-	-
Russia	-	-	-	-	-	CHY	-	-	-	-		-	-	-	+
Mongolia	-	-	-	-	-	-	-	-	-	-				-	+
South Africa	-	-	-	-	-	-	-	-	-	-			-	-	-
UK	-	-	-	-	-	-	-	-	-	-	-		-	-	-
Eire	-	-	-	-	-	-	-	-	-	-	-		-	-	-
France	-	-	-	-	-	-	-	-	-	-	-		-	-	-
Netherlands	-	-	-	-	-	-	-	-	-	-	-		-	-	-
Afghanistan		+	+				ME-SA	+							+
Pakistan	+	+	+		ME-SA	+		ME-SA	ME-SA				ME-SA	ME-SA	-
Bulgaria	-	ME-SA	-	ME-SA	-	-	ME-SA	-	-	-	-			-	-
Greece	-	-	-	-	ME-SA	-	ME-SA	-	-	-	-	-	-	-	-
Bangladesh	+	+	+	+	+	+	ME-SA	+	+	+				-	
Armenia							ME-SA	+	-	-					
Azerbaijan							+	+	-	-					
Georgia							+	ME-SA			+			-	
Hong Kong	CHY	CHY	CHY	CHY	CHY	CHY	CHY	CHY	CHY	CHY	CHY	CHY	CHY	CHY	CHY
Indonesia	-	-	-	-	-	-	-	-	-	-	-	-	-	-	-
Kazakhstan								-	+	+	+				
Kyrgyzstan		ME-SA	-						+	-					
North Korea	-	-	-	-	-	-	-	-	-	-	-	-	-	-	-
Oman	+	ME-SA	+	+	+	+						ME-SA		-	-
Philippines	-	-	-	-	CHY	CHY	CHY	CHY	CHY	CHY	CHY	CHY	CHY	CHY	CHY
Tajikistan					ME-SA	-	-		-	-	+			-	
Turkmenistan						-	-	+	+	+					
Uzbekistan		ME-SA	-	-	-	-	-	-	-	-	-				

*Compiled from the Food and Agriculture Organization/World Health Organization/Organization for Animal Health Yearbooks and the records of the World Reference Laboratory for FMD.  †Abbreviations: solid boxes, PanAsia virus isolated; blank boxes, unknown disease situation; +, FMD type O present (genotype not known); -, no FMD type O reported;  CHY, Cathay topotype; ME-SA, Middle East-South Asia topotype (other than PanAsia strain); SEA, Southeast Asia topotype.
